# Complete Recovery of Renal Function among Obstetric Patients with Acute Kidney Injury at a Tertiary Care Hospital: A Descriptive Cross-Sectional Study

**DOI:** 10.31729/jnma.7135

**Published:** 2021-12-31

**Authors:** Rakina Bhansakarya, Gehanath Baral, Shailendra Shrestha, Shanti Subedi, Sita Ghimire, Prajmi Shrestha, Amar Nath Chaudhary

**Affiliations:** 1 Department of Obstetrics and Gynaecology, Nobel Medical College and Teaching Hospital, Biratnagar, Nepal; 2 Department of Nephrology, Nobel Medical College and Teaching Hospital, Biratnagar, Nepal

**Keywords:** *acute kidney injury*, *dialysis*, *preeclampsia*, *sepsis*

## Abstract

**Introduction:**

Acute kidney injury is a rare complication of pregnancy and is associated with high maternal morbidity and mortality. Obstetric factors associated with it are preeclampsia/eclampsia, sepsis, hemorrhage and dehydration. Here, we aim to find out the prevalence of complete recovery of renal function among obstetric patients with acute kidney injury.

**Methods:**

This is a descriptive cross-sectional study conducted in a tertiary care hospital from 1st July 2020 to 30th June 2021 where obstetric patients who had developed acute kidney injury were included and followed till 6 weeks of diagnosis. Ethical approval was obtained from Institutional Review Committee of Nobel Medical College and Teaching Hospital (Reference number 437/2020). The convenience sampling method was used. Data entry and analysis were done using Statistical Package for Social Sciences version 21. Point estimate at 95% Confidence Interval was calculated along with frequency and proportion for binary data.

**Results:**

Out of total 66 obstetric patients with acute kidney injury, 45 (68.2%) (57-79.3 at 95% Confidence Interval) had complete recovery of renal function. Rate of renal function recovery in Stage 1, Stage 2 and Stage 3 acute kidney injury were 19 (90%), 19 (86%) and 7 (58%) respectively. The most common causes of acute kidney injury were Preeclampsia/eclampsia 18 (40%), sepsis 23 (28.8%) and hemorrhage 10 (22.2%).

**Conclusions:**

The prevalence of complete recovery in obstetric patients with acute kidney injury was similar to findings from other studies done in similar settings.

## INTRODUCTION

Acute kidney injury (AKI) refers to an abrupt decrease in kidney function with retention of nitrogenous waste products. The incidence of acute kidney injury has decreased in recent years due to improved maternal health care.^1^ However, it is still a major cause of maternal morbidity and mortality in developing countries.^2^

According to Wiles, et al. AKI complicates 1.4% admission in the United Kindgom (UK) whereas in developing countries, it is 4-15%.^2,3^ The current data do not accurately state the incidence of acute kidney injury because of diverse defining criteria of AKI in pregnancy. The value of serum creatinine is less than normal range in pregnancy and level more than 1.02mg/dl is diagnostic of AKI.^4^ AKI in pregnancy is associated with septic abortion in early pregnancy and preclampsia/eclampsia and hemorrhage in late pregnancy.^1^ In the past, AKI was considered to be a reversible pathology, but recent studies have shown otherwise.

The aim of this study is to prevalence of complete recovery of renal function among obstetric patients with acute kidney injury in a tertiary care hospital.

## METHODS

This was a descriptive cross-sectional study conducted in Nobel Medical College and Teaching Hospital from 1st July 2020 to 30th June 2021 among obstetric patients who had developed acute kidney injury. Ethical clearance was taken from Institutional Review Committee of Nobel Medical College and Teaching Hospital (Ref IRC-NMCTH 437/2020). Obstetric patients (pregnant and puerperal women) who presented with features of acute kidney injury characterized by oliguria (<400ml of urine in 24 hours) or rise in creatinine >1.02mg/dl were included in the study. Patients with preexisting renal disease or renal insufficiency before pregnancy were excluded from the study. Convenience sampling was done and the sample size was calculated as,

n = Z^2^ × p × q / e^2^

  = (1.96)^2^ × (0.89) × (1-0.89) / (0.08)^2^

  = 59

Where,

n= minimum required sample size
Z= 1.96 at 95% Confidence Interval (CI),
p= prevalence of complete renal recovery in acute kidney injury in pregnancy, 89%^5^
q= 1-p
e= margin of error, 8%

Minimum sample calculated was 59 and after taking a 10% non-response rate, a total of 66 eligible cases were enrolled into the study. Written informed consent was taken. Data were collected on a structured questionnaire and patient was followed daily till discharge and in six weeks' time.

Variables studied were age, gravida, maximum serum creatinine level, need of dialysis, serum creatinine at discharge, serum creatinine at 6weeks follow-up and associated obstetric complications.

Acute kidney injury (AKI) was defined and classified according to Kidney Disease: Improving Global Outcome (KDIGO) criteria based on changes in serum creatinine or changes in urine output, or both, and it was categorized in stage 1 (increase in creatinine level by 1.5 times the baseline or urine output <0.5ml/kg/hr for 6-12hours), stage 2 (increase in serum creatinine level by 2 times the baseline or urine output <0.5/ml/kg/hr for ≥12hour) and stage 3 (increase in serum creatinine level by 3 times the baseline or creatinine level ≥4mg/dl or urine output <0.3ml/kg/hr for 24hours or anuria for ≥12hours).^6^

Data were entered in Microsoft Excel and imported to Statistical Package for the Social Sciences (SPSS) version 21 for analysis. Data were tabulated in frequency tables. Categorical variables were expressed as frequencies whereas continuous variables were expressed as mean and standard deviation. Point estimate at 95% CI was calculated.

## RESULTS

The prevalence of complete renal recovery in acute kidney injury was 45 (68.2%) (57-79.3 at 95% CI). Further sub-analysis were done in them and which showed the patient's age ranged from 18 to 35 years (24.1±4.69) and gravidity ranged from 1 to 5 (2.85 ±0.88). Among them, acute kidney injury occurred most commonly in third trimester 20 (44.4%) followed by postpartum period 15 (33.3%), first trimester 7 (15.5%) and second trimester 3 (6.6%). Preclampsia/Eclampsia was the most common cause of acute kidney injury in pregnancy 18 (40%) followed by sepsis 13 (28.8%) and hemorrhage 10 (22.2%). Other etiology of acute kidney injury was dehydration 2 (4.4%) and urinary tract infection/obstructive uropathy 2 (4.4%). Trimester wise distribution of causes of AKI is shown in Table 1.

**Table 1:** Gestational age specific etiology of completely recovered acute kidney injury.

Etiology	Preeclampsia/Eclampsia n (%)	Sepsis n (%)	Hemorrhage n (%)	Dehydration n (%)	Urinary tract infection/Obstructive uropathy n (%)
First trimester	0 (0)	3 (43)	2 (28.5)	2 (28.5)	0 (0)
Second trimester	0 (0)	2 (66.6)	1 (33.3)	0 (0)	0 (0)
Third trimester	15 (75)	1 (5)	3 (15)	0 (0)	1 (5)
Puerperium	3 (20)	7 (46.6)	4 (26.6)	0 (0)	1 (6.6)
Total	18 (40)	13 (28.8)	10 (22.2)	2 (4.4)	2 (4.4)

**Figure 1:**
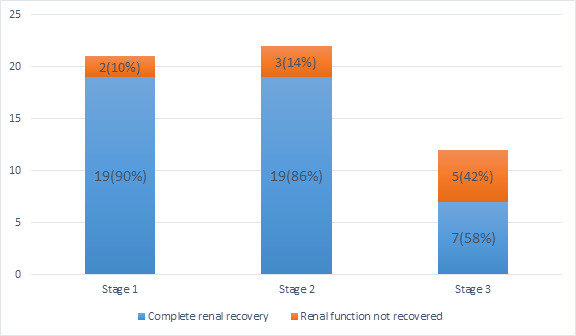
Renal function recovery in different stages of acute kidney injury.

In the patients with complete renal recovery, the rate of renal function recovery decreased significantly with increasing stage of AKI; 19 (90%) of Stage 1, 19 (86%) of Stage 2 and 7 (58%) of Stage 3 (Figure 1).

Of these patients, 19 (54.2%) had intramural deliveries whereas 16 (45.7%) had extramural deliveries. Moreover, only 8 (17.7%) cases were booked cases. Among deliveries, 15 (42.8%) and 20 (57.2%) were preterm and term deliveries respectively. Of these, 16 (45.7%) were vaginal deliveries and 19 (54.3%) were cesarean section.

The average creatinine was 2.32± 1.42mg/dl with minimum and maximum values of 1.1mg/dl and 6mg/dl respectively. Dialysis was needed in 4 (8%) cases. Stage wise distribution of obstetric factors associated with acute kidney injury (Table 2).

**Table 2:** Gestational age specific stages of completely recovered AKI.

	Stage 1 n (%)	Stage 2 n (%)	Stage 3 n (%)
First trimester	1 (14.3)	4 (57.1)	2 (28.6)
Second trimester	0	2(66.6)	1(33.4)
Third Trimester	10 (50)	7(35)	3 (15)
Puerperium	8 (53.3)	6 (40)	1 (6.6)

There were 2 (4.4%) cases of intrauterine fetal death and 1 (2.2%) cases of neonatal death.

## DISCUSSION

Pregnancy related acute kidney injury has serious risk of maternal morbidity and mortality. Complete recovery of renal function occurred in 68.2% patients of AKI with lower recovery rate for higher stages of AKI. Study by Prakash et al also agreed to our finding which noted complete renal recovery in 69.4%.^7^ However, Goplani et al has noted renal recovery in only 54% while other studies has reported complete renal recovery as high as 89% cases.^2,8^

In the present study, preeclampsia/eclampsia was the most common cause of AKI followed by sepsis (40% and 28.8%). This finding agrees with that of Wang, et al. (49%) and Prakash, et al. (35.25%) which also showed that most common cause of AKI was due to preeclampsia.^7,9^ Similar to our study, Ansari, et al. also reported sepsis in 31% cases.^10^

The majority of women did not require hemodialysis and were managed conservatively. Dialysis was required only in 8% cases and all of them were Stage 3 AKI and this finding is similar to other studies.^1,2^

AKI was more common in third trimester (44.4%) closely followed by postpartum (33.3%) and similar finding were noted in a study in Morocco whereas, study by Ansari, et al. noted AKI to be more common in postpartum period (75.6%).^1,10^

The mean age of patient and gravidity were 24.1±4.69 years and 1 to 5 (2.85±0.88) and similar finding were reported in other studies.^1,9^

AKI was found to exist with increased perinatal complication like premature delivery (42.8%) and perinatal mortality (8.5%) in our study and similar finding were seen in study by Arora, et al.^8^ Other studies have noted high perinatal mortality ranging from 38.8% to 55%.^9,11^

High number of cases of acute kidney injury in pregnancy in this hospital is due to late referral from periphery hospitals. Poor prenatal care, lack of well-equipped hospital in periphery, difficult road transport leads to delay in reaching tertiary hospital and in management followed by increasing severity of AKI. Majority of cases (82.3%) of acute kidney injury were unbooked cases and 45.7% cases had extramural deliveries which shows the need for early assessment and detection of morbidity for proper pregnancy care. Efforts are required to improve the coverage of prenatal care, especially in peripheral areas.

In study by Prakash, et al. renal cortical necrosis was diagnosed in patients with incomplete recovery of renal function and its incidence was noted to have decreased from 17% in 1982-1991 to 2.4% in 2000s.^9^ But in the present study, this factor was not explored.

The other limitation of this study was the small sample size and lack of long-term follow-up of the patient.

## CONCLUSIONS

The prevalence of complete recovery in obstetric patients with acute kidney injury was similar to other studies. The higher stage of AKI was seen with low recovery pattern such that least complete renal recovery occurred in Stage 3 AKI. This study is limited due to short follow-up so further studies are needed to understand the long-term outcome of AKI in pregnancy. Adequate antenatal care is needed to detect and prevent AKI and reduce poor long-term morbidity associated with this condition.
